# Psychometric Properties of a Short Version of the Impulsiveness Questionnaire UPPS-P in a Brazilian Adult Sample: Invariance for Effects of Age, Sex and Socioeconomic Status and Subscales Viability

**DOI:** 10.3389/fpsyg.2018.01059

**Published:** 2018-06-27

**Authors:** Sabine Pompeia, Luanna Maristella Inacio, Rafaella Sales de Freitas, Gislaine Valverde Zanini, Leandro Malloy-Diniz, Hugo Cogo-Moreira

**Affiliations:** ^1^Departamento de Psicobiologia, Universidade Federal de São Paulo, São Paulo, Brazil; ^2^Departamento de Saúde Mental, Universidade Federal de Minas Gerais, Belo Horizonte, Brazil; ^3^Departamento de Psiquiatria, Universidade Federal de São Paulo, São Paulo, Brazil

**Keywords:** impulsive behavior, self-control, affect, attention, risk-taking, sex factors, socioeconomic factors, age factors

## Abstract

Five different facets or domains of impulsivity (lack of Perseverance, lack of Premeditation, Sensation Seeking, Positive and Negative Urgency) have been detected in undergraduate students by means of a short, 20-item version of the Impulsive Behavior Scale UPPS-P. The present cross-sectional study examined the psychometric properties of a Brazilian version of this short scale (SUPPS-P) in a non-clinical sample of 510 individuals with a larger age range (10–72 years) and from varying socioeconomic strata (SES). We also investigated: (a) differential item functioning according to age, sex and socioeconomic status; (b) whether these demographic factors affected participants’ responses (population heterogeneity); and (c) if using scores directly derived from respondents’ answers (raw scores) reflected the 5 distinguishable impulsiveness domains out of the structural equation modeling environment (bifactor model). We showed that the short UPPS-P version replicated factor structures, internal consistency across domains and inter-scale correlations found in prior studies, and confirmed the psychometric separability of the 5 impulsiveness domains. Only three out of the 20 items showed differential item functioning. Higher Positive and Negative Urgency and lack of Premeditation were reported by men and impulsiveness decreases with age in all domains except lack of Premeditation. SES did not influence results. The viability of using raw scores to assess the five domains was not confirmed via bifactor modeling. The use of a general composite score was psychometrically acceptable. We conclude that, in the structural equation modeling environment, the SUPPS-P is a reliable instrument to assess multiple impulsivity domains in non-clinical community samples in different cultural settings. However, out of this statistical environment, viability was only found for a general factor of impulsivity.

## Introduction

Impulsive behavior involves acting without delay, reflection, voluntary direction or control in response to stimuli (Medical Subject Headings, MeSH Unique ID: D007175^1^). Although impulsivity (or impulsiveness) is considered by some as a unitary construct, it has been proposed that it encompass various facets or domains which vary among non-clinical populations (e.g., Whiteside and Lynam, 2001; Whiteside et al., 2005; Smith et al., 2007) and individuals with psychopathologies (see Berg et al., 2015).

A widely used (see Berg et al., 2015) instrument that assesses overlapping but distinguishable impulsive domains is the self-report Impulsive Behavior scale UPPS (Whiteside and Lynam, 2001; Whiteside et al., 2005). The domains that this scale reflected factors, obtained in exploratory factor analysis, that aggregated items/questions from various valid and reliable questionnaires that measure traits associated to impulsivity (Whiteside and Lynam, 2001; Whiteside et al., 2005). Each domain has been found to be differently associated to behavior in several psychopathological conditions such as alcohol/substance abuse, attention deficit hyperactivity disorder, eating disorders, as well as traits found in non-clinical samples, such as variations in aggressiveness, self-discipline, academic performance, anxiety and depressive symptoms, and risk taking (e.g., gambling, engaging in antisocial and illegal activities) (e.g., see Whiteside et al., 2005; Cyders and Smith, 2007; Smith et al., 2007; Berg et al., 2015).

UPPS is an acronym composed of letters that represent each of its impulsiveness domains: (1) Negative Urgency, the tendency to commit rash or regrettable actions as a result of negative affect; (2) (lack of) Premeditation, the tendency not to reflect on the consequences of one’s actions; (3) (lack of) Perseverance, or difficulty in staying focused on hard of tedious tasks; and (4) Sensations Seeking, the tendency to seek new and exciting experiences. A fifth domain, Positive Urgency (“-P”), or the tendency to experience strong impulses when in unusually positive mood (see Cyders and Smith, 2007; Berg et al., 2015), was later added to the scale, forming an instrument named UPPS-P.

The UPPS-P is a relatively long scale containing 59 items, a somewhat inappropriate characteristic if testing time is short and for populations who tire and become easily distracted, such as youngsters and people with low socioeconomic status/schooling. To circumvent these limitations, reduced versions of the scale have been proposed, such as the one by Cyders et al. (2014), called short UPPS-P (SUPPS-P). To build this scale, items with highest corrected item-total correlation for each domain in the full UPPS-P version were initially selected. Redundant items (i.e., those with inter-item correlations greater than 0.50 with the already selected item) were discarded and then the next most correlated item within the domain was selected. The procedure was repeated until four items per domain were selected, totaling 20 items (Cyders et al., 2014).

Compared to the full UPPS-P, the SUPPS-P of Cyders et al. (2014) was completed much faster and maintained comparable factor structure, internal consistency and subscale inter-correlations, with only a minimal loss of shared variance (Cyders et al., 2014). Such a short scale that allows the identification of separable impulsivity traits is of great interest for research and clinical purposes worldwide.

There is another short, 20-item version of the UPPS-P that was proposed by Billieux et al. (2012) (translated into Spanish: Cándido et al., 2012; Italian: D’Orta et al., 2015; and Arabic: Bteich et al., 2017). To build this short scale the authors followed a different approach to that used by Cyders et al. (2014): they chose the four items with the highest factorial loads in their respective domains. As explained in Cyders et al. (2014), although this may preserve the reliability of the reduced scale by eliminating items with more error variance, it can increase redundancy and in this way may reduce content validity.

The SUPPS-P (Cyders et al., 2014) and the publications derived from Billieux et al.’s (2012) work studied the psychometric properties of the scales mostly in highly educated young adults from developed nations. It would be of interest to determine whether this type of scale could be used in populations with different cultural and demographic characteristics. After all, various facets of impulsiveness are affected by respondents’ age, sex, socioeconomic status (e.g., Costa et al., 2001; Steinberg et al., 2008; Reimers et al., 2009; Cross et al., 2011; Chamorro et al., 2012; Cyders, 2013) and, possibly reflect differences in culture, genetics, biological and environmental backgrounds (Kacen and Lee, 2002; Bezdjian et al., 2011; Cross et al., 2011; Chamorro et al., 2012). For example, in the United States, higher impulsiveness is associated with being born in that country, non-Hispanic white, never married and aged 18–29, while lower impulsiveness is found in people with high educational attainment and income (Chamorro et al., 2012). It is therefore not unreasonable to suppose that varying demographic characteristics could influence the factor structure of the SUPPS-P, possibly making it inadequate in samples other than undergraduate students from high-income industrial countries. In effect, the factor structure of the SUPPS-P in undergraduate students from Iran (Shokri and Sanaeepour, 2016), a country with different demographics to those in the United States, needed some corrections to be comparable to the 5 factor model solution found by Cyders et al. (2014).

Hence, in order to determine the extent to which Cyders et al.’s (2014) SUPPS-P is useful in populations that are not highly educated young adults from fully developed nations, the present study investigated whether a translated version of the SUPPS-P into Portuguese would have adequate psychometric properties in a Brazilian non-clinical community sample. We also investigated the effects of age, sex and socioeconomic status on the way participants responded to the scale items (invariance testing) and on the latent traces in the 5 domains of impulsivity.

Because we included in our sample many under-aged individuals who had not reached their maximum schooling levels, participants’ schooling was not controlled for. Instead, we used parental education as a proxy for socioeconomic status (see Sirin, 2005) because it reflects home and school environments while their progeny is growing up, which influences biopsychosocial trajectories of development (see Cohen et al., 2010). In adults worldwide, schooling tends to remain stable over time, as do income and occupation (see Sirin, 2005), so parental schooling is unlikely to change. Furthermore, as there is not much intergenerational social mobility in Brazil (Ribeiro, 2014), this measure can indicate indirectly, to some extent, participants’ present socio class.

Lastly, we assessed the practical utility of raw scores (directly derived from participants’ responses, and not latent variables) to indicate the separability of the 5 domains of the UPPS-P. In other words, we studied if it is reliable and viable to use such scores out of the structural equation modeling environment (bifactor modeling).

## Materials and Methods

### Participants

This study involved a Portuguese-speaking non-clinical community sample that either responded to a translated version of the SUPPS-P available online or provided responses in person (young population over the age of 9 years, with legal guardian agreement).

### Procedure

This cross-sectional study was conducted according to international ethical guidelines and the Brazilian National Heath Council ethical resolution (Resolução 466/12). It was approved by the Ethic Committee of the Universidade Federal de São Paulo (UNIFESP) (#1.976.055; #2.001.042). All participants and legal guardians, when applicable, provided informed consent and/or assent. A demographic questionnaire and the SUPPS-P (see below) were made available online in the platform Google Forms for 5 months. Recruitment of respondents was made through social media. For the under-aged participants, the same questionnaires were printed out and handed to minors in a waiting room at an adolescent clinic at UNIFESP during the same period. Data other than cited below were also collected and results pertaining to them will be reported elsewhere.

### Demographic Questionnaire

We enquired about participants’ age, sex, and schooling of their male and female parents (or corresponding guardians with these roles) in seven strata (ordinal variable) according to the Brazilian educational system: 1 (incomplete basic schooling, which lasts 8 or 9 years depending on participants’ age); 2 (complete basic schooling); 3 (incomplete high school, which lasts 3 years); 4 (complete high school); 5 (incomplete tertiary education, which usually lasts 4 years); 6 (complete tertiary education); or 7 (any sort of post-graduate training).

### SUPPS-P

The items from the SUPPS-P (Cyders et al., 2014) in Portuguese pertaining to all impulsiveness domains except Positive Urgency were obtained from the research team that adapted the UPPS for use in Brazil, which was shown to display adequate psychometric properties (Nogueira et al., 2013; Sediyama et al., 2017). These researchers also provided translations of the Positive Urgency items, which are currently under validation. Items of the scale are affirmations. Respondents are asked to report the extent to which they agree with each statement on four-point Likert scales ranging from 1 (agree strongly) to 4 (disagree strongly). In our version, higher scores in the domains Perseverance and Premeditation indicated higher impulsivity, while for the domains Sensation Seeking, Positive and Negative Urgency, lower scores indicated more impulsivity.

### Statistical Analysis

The analysis was undertaken in various steps, using Mplus 8.0 (Muthén and Muthén, 1998–2017).

Firstly, we tested three models specified by Cyders et al. (2014) using Confirmatory factor analyses (CFA) under weighted least squared mean-variance estimator (WLSMV) due to the four-point Likert structure of SUPPS-P. The following models were investigated: Model 1 included all 20 SUPPS-P items loading onto a single general impulsivity factor. Model 2 included five latent traits corresponding to the five SUPPS-P domains, each with four items. Model 3 is specified in a second-order, hierarchical structure, having two higher order latent variables (see Cyders and Smith, 2007): 1) Emotion-based Rash Action (formed by Negative and Positive Urgency first order factors); 2) Deficits in Conscientiousness (formed by lack of Perseverance and of Premeditation first order factors). Sensation seeking was kept as a first order factor. To compare the nested models 2 and 3 we used the Delta CFI (Cheung and Rensvold, 2002), which indicates worsening in model fits when values are greater than 0.002 (Meade et al., 2008). This is a more stringent approach to compare models than the use of χ2 (Meade et al., 2008) applied by Cyders et al. (2014).

We tested model invariance through the Multiple Indicators Multiple Causes (MIMIC) method for the covariates sex, age, mother and father’s level of education. This was done under Model 2, which had a good fit and informs on the separability of all 5 domains (see Results and Discussion for details on the reasons for this selection). We followed the two basic steps for MIMIC modeling (Brown, 2015). First, a measurement model was established using the full sample (in our case, in Model 2). Then, the direct effects of the covariates on the factors and the indicators (items) were evaluated. When a significant direct effect of the covariate on the factor is found, it indicates population differences or heterogeneity (i.e., the factor means are different at different levels of the covariates). On the other hand, a significant direct effect (i.e., modification indices > 4) of the covariate on an indicator of a factor (i.e., the UPPS-P items) represents Differential Item Functioning (DIF). This means that responses to specific item are different at different levels of the covariate. As described in Brown (2015, p.282) and here tested “[MIMIC] is frequently evaluated in an exploratory fashion.” We fixed all direct effects between sex, age, mother and father’s schooling and the five-factors correlated solution indicators to zero. We then inspected modification indices to determine whether relevant direct effects were present. To evaluate the reliability (called rho, *ρ*) of the five factors under the specifications of Model 2, we used the factor loadings and residual variances from the CFA’s Model 2 as described by Joreskog (1971) and Dillon and Goldstein (1984).

To assess the reliability and viability of SUPPS-P’s subscales using raw scores derived directly from participants’ answers, and not from latent traces in the structural equation modeling environment, we ran another model (Model 4). This bifactor structural model (also called general-specific model) specifies that the covariance among a set of item responses can be accounted for by two main sources of information. The first is a single general factor that reflects the common variance to all scale items (in our case, the general concept of impulsivity); the second source of information derives from group factors (the five domains of impulsivity) that reflect additional common variance among clusters of items with similar content. It is assumed that the general and group factors are all orthogonal (i.e., not correlated) (**Figure 4**). Under a bifactor model, different indices can be computed: (a) *Coefficient omega* (*Lucke’s*ω) (Revelle and Zinbarg, 2009; Reise, 2012; McDonald, 2013), which is a reliability estimate based on factorial model that estimates the proportion of the observed variance in the total score attributed to all sources of common variances; (b) *Coefficient omega hierarchical* (ω_h_) (Reise et al., 2011; Rodriguez et al., 2016a), which is a reliability index that judges the degree to which the composite scale scores are interpretable as a measure of a single common factor. – The coefficient omega hierarchical is computed by dividing the squared sum of the factor loadings on the general factor (*model estimated*) by the variance of total scores -; (c) *Coefficient omega subscale* (ω_s_) (Reise et al., 2011; Rodriguez et al., 2016a) reliability and viability estimate for a residualized subscale, controlling for that part of the reliability due to the general factor (i.e., the percentage of the subscale score variance attributable to a specific group factor of items after removing the reliable variance due to the general factor). – This index reflects the reliability of a subscale score after controlling for the variance due to the general facto r -; and (d) *Explained common variance* (ECV), which is the percentage of common variance explained by the general factor. This is a type of unidimensionality index which is directly related to the relative strength of the general factor. It can be defined as the ratio of the explained variance by the general factor divided by the variance of the general and specific factors. Details of these calculations can be found in (Rodriguez et al., 2016a,b).

For *Lucke’s omega* (ω), *Coefficient omega hierarchical* (ω_h_), and *Coefficient omega subscale* (ω_s_), scores higher than 0.8 indicate a strong relationship between the latent variable and item scores. An ECV higher than 0.70 indicates that the instruments should be treated as essentially unidimensional, as a single common factor (Rodriguez et al., 2016a,b).

## Results

The sample was composed of 528 participants. There were incomplete SUPPS-P data from 9 under-aged and 9 adult volunteers so their data were excluded from the analyses. The sample used in the models was thus of 510 individuals (27% of whom were male) aged 10–72 years (mean age = 25.4, *SD* = 12.3 years). There were 160 participants under the age of 18, 210 between the ages of 18 and 29 years, 105 aged 30–49 years, and 35 were aged 50 or older. Schooling of parents (the measure of socioeconomic status) ranged from strata 1 to 7, with a mean score (mean = 4.3 years, SD ± 2.0) that corresponds to having completed high school. The sample was varied in this respect as there was a minimum of 23 cases in each of the 7 mother or father schooling stratifications. Ten participants reported not having had a person who acted as a mother and 39, as a father.

### Structural Equation Modeling Following Cyders et al. (2014)

**FIGURE 1:**
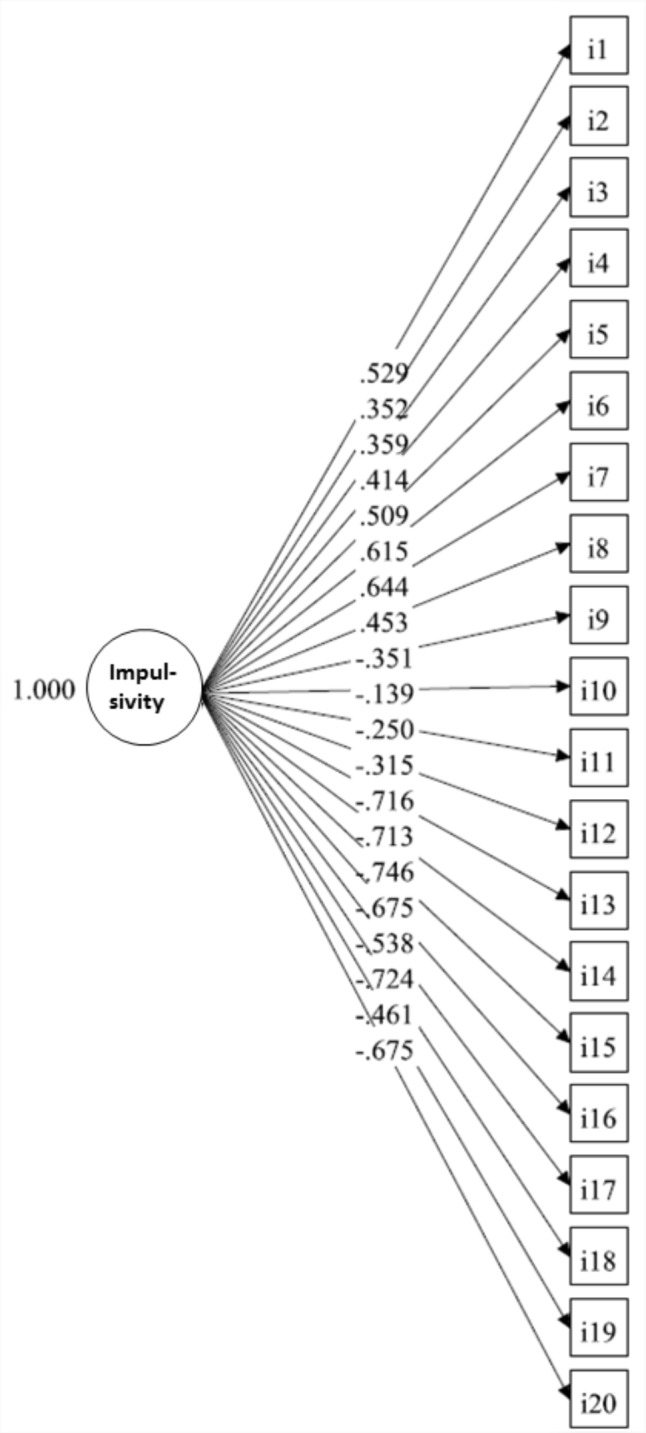
Model 1, including all 20 items of the Short Impulsive Behavior Scale SUPPS-P loading onto a single “impulsivity” factor. N.B. Individual items (i1–i20) from Cyders et al. (2014) are identified in boxes. The first 8 items correspond to the domains Perseverance and Premeditation (4 items each, in order) for which lower scores indicated higher impulsivity. The following items refer to Sensation Seeking, Positive Urgency and Negative Urgency (Neg), for which higher scores indicate more impulsiveness. Values on single headed arrows indicate factor loadings.

**FIGURE 2:**
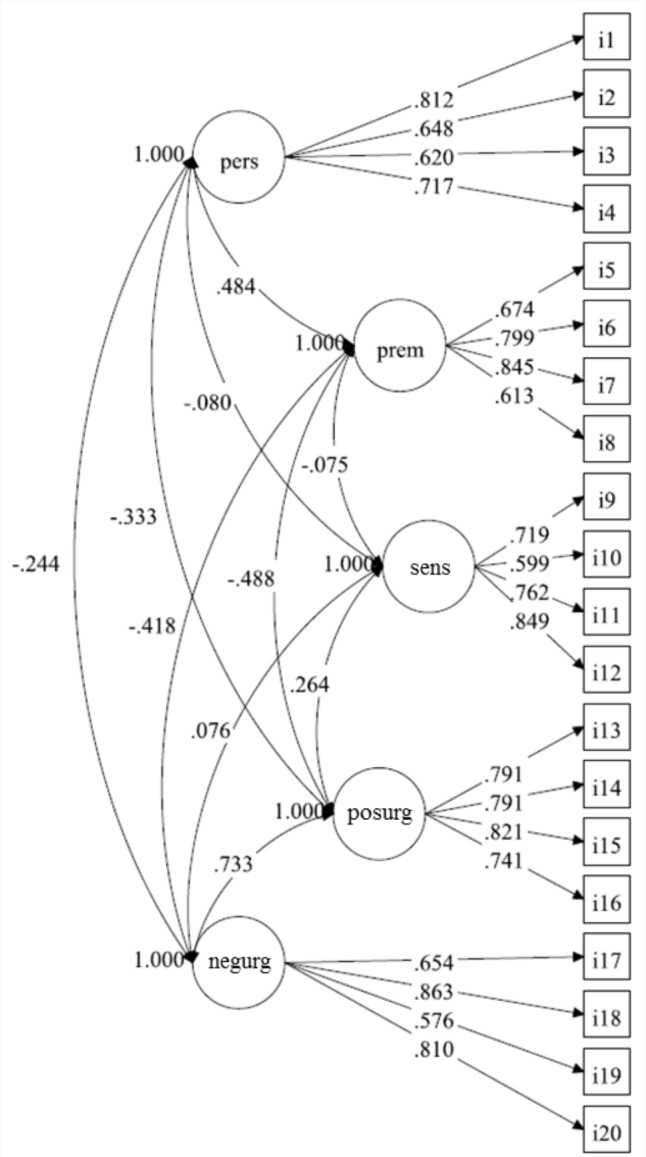
Five-factor model (Model 2) of the Short Impulsive Behavior Scale (SUPPS-P), in which groups of four items (i) load onto their specific domains. N.B. individual items (i1–i20) are identified in boxes and impulsive domains, in circles. Higher scores in the domains Perseverance (pers) and Premeditation (prem) indicate lower impulsivity, while higher scores indicate more impulsiveness in Sensation Seeking (sens), Positive Urgency (posurg), and Negative Urgency (negurg). Values on double headed arrows indicate correlations among domains (those with *r* > 0.09 had *p* < 0.05); values on single headed arrows indicate factor loadings.

**FIGURE 3:**
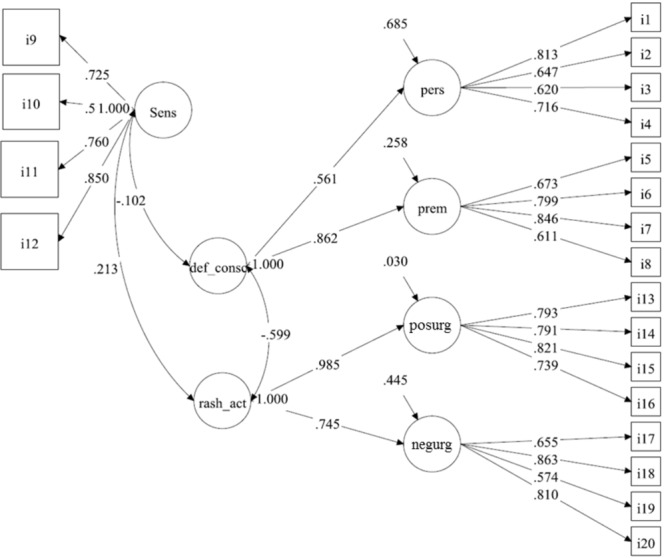
Model 3 on data from the Short Impulsive Behavior Scale (SUPPS-P), with a second-order hierarchical structure with two higher order latent variables (see Cyders and Smith, 2007): (1) Emotion-based Rash Action (formed by Negative and Positive Urgency first order factors; rash_act); (2) Deficits in Conscientiousness (formed by lack of Perseverance and of Premeditation first order factors; def_consc). Sensation seeking (Sens) was kept as a first order factor. N.B. individual items (i1–i20) are identified in boxes and impulsive domains, in circles. Lower scores in the domains Perseverance (pers) and Premeditation (prem) indicate higher impulsivity, while higher scores indicate more impulsiveness in Sensation Seeking (Sens), Positive Urgency (posurg) and Negative Urgency (negurg). Values on double headed arrows indicate correlations among domains; values on single headed long arrows indicate factor loadings. Short arrows indicate residual variance.

Regarding the models proposed by Cyders et al. (2014), with no correction for demographic characteristics, we found that Model 1 (one-factor model), with all 20 SUPPS-P items loading onto a single “impulsivity” factor, fit data poorly [χ^2^_(170)_ = 2884.743, *p*-value < 0.001, RMSEA = 0.176 (90%CI = 0.171 to 0.182), CFI = 0.567, TLI = 0.516] (**Figure 1**). In contrast, adequate fits were obtained for Model 2 (**Figure 2**), with five first-order correlated latent factors corresponding to the five SUPPS-P domains [χ^2^_(160)_= 536.059, *p*-value < 0.001, RMSEA = 0.068 (90%CI = 0.061–0.074); CFI = 0.940; TLI = 0.929], and Model 3 (**Figure 3**), the second order factor model with 2 s order factors (emotion-based rash action and deficits in conscientiousness, and Sensation Seeking as a first order factor) [χ^2^_(163)_ = 527.974, *p*-value < 0.001, RMSEA = 0.066 (90%CI = 0.060–0.072), CFI = 0.942, TLI = 0.932]. Low inter-correlations between the majority of the domains were found in Models 2. The two pairs of domains with the highest correlations formed the 2 s order factors in model 3. Adding this restriction (e.g., second order factor) in Model 3, when compared to model 2, did not worsen the fit indices (ΔCFI = 0.942 minus 0.940 = 0.002). Under Model 2 structure, the reliability of the five factors (ρ) were: ρ of Negative Urgency = 0.820; ρ of Perseverance = 0.794; ρ of Premeditation = 0.825; ρ of Sensation Seeking = 0.824; ρ of Positive Urgency = 0.866. *ρ* were not assessed for Model 3 as they are not appropriate for hierarchical models.

### Measurement Invariance (MIMIC model)

Here, again we focused on Model 2 (see more details about this in the Discussion section). We identified only three items with DIF related to sex. Males had a higher probability of disagreeing with the statement “Unfinished tasks really bother me” (Premeditation) and agreeing with “I would like to learn to fly an airplane” (Sensation Seeking) and “Others are shocked or worried about the things I do when I am feeling very excited” (Positive Urgency) than females. Moreover, the latter item also exhibited a DIF in terms of age (i.e., the older the participant, the higher the probability of disagreeing with this statement). No items with DIF were associated to parental schooling (measure of socioeconomic status).

### Population Heterogeneity

Regarding latent outcomes using Model 2, we found an effect of sex on Premeditation (beta = -0.134; *p* = 0.01), Positive (beta = 0.115; *p* = 0.02) and Negative (beta = 0.123; *p* = 0.02) Urgency, indicating that males were more impulsive on these domains. Older ages were associated with lower impulsiveness in all domains except Premeditation [Perseverance (beta = -0.185, *p* < 0.001); Sensation Seeking (beta = 0.178, *p* < 0.001); Positive Urgency (beta = 0.178, *p* < 0.001); and Negative Urgency (beta = 0.201, *p* < 0.001)]. Parental schooling had no effects (*p*-values > 0.17).

### Bifactor Model and Scale Reliability and Viability When Using Scores Directly Obtained From Participants’ Answers

**FIGURE 4:**
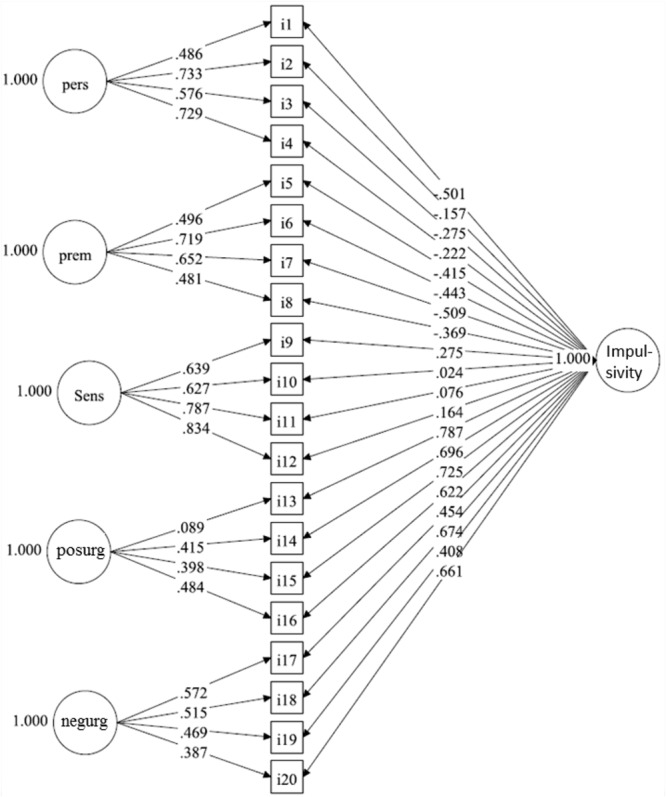
Bifactor structural model (Model 4) on data of the Short Impulsive Behavior Scale (SUPPS-P), which specifies that the covariance among a set of item responses can be accounted for by two main sources of information: (1) a single general factor of impulsivity that reflects the common variance among all scale items; (2) group factors (the five domains of impulsivity) that reflect additional common variance among clusters of items with similar content. N.B. Individual items (i1–i20) are identified in boxes and impulsive domains, in circles. Lower scores in the domains Perseverance (pers) and Premeditation (prem) indicate higher impulsivity, while higher scores indicate more impulsiveness in Sensation Seeking (Sens), Positive Urgency (posurg), and Negative Urgency (negurg). Values on single headed arrows indicate factor loadings.

Model 4 (**Figure 4**), the less restrictive model (the bifactor solution), returned the following fit indices: [χ^2^_(150)_ = , *p*-value < 0.001, RMSEA = 0.062 (90%CI = 0.055 to 0.068), CFI = 0.953, TLI = 0.941]. Based on this model, we computed a model-based reliability estimate for each of the SUPPS-P subscales using Lucke’s omega, applying it to one domain at a time: ω of Negative Urgency = 0.828; ω of Perseverance = 0.797; ω of Premeditation = 0.824; ω of Sensation Seeking = 0.829; ω of Positive Urgency = 0.875. Although these reliabilities were good, they were considerably lowered when the effects of the general impulsivity factor was removed in Model 4, with the exception of the domain Sensation Seeking [this evaluation was conducted via *Coefficient omega subscale* [ω_s_]: ω(s)_Perseverance_ = 0.659, ω(s)_Premeditation_ = 0.533, ω(s)_Sensation Seeking_ = 0.801, ω(s)_Positive Urgency_ = 0.169, and ω(s)_Negative Urgency_ = 0.363]. Other indices derived from the bifactor model were: EVC = 0.404, ωH = 0.673, Lucke’s ω for the whole scale = 0.917. From ω_H_, we found that 67.3% the variance in the unit-weighted total scores could be attributed to the differences between participants in the general impulsivity factor. The square root of ω_H_ (82.03%) indicated a very strong correlation between the general impulsivity factor and the observed raw scores. Only 8.3% of variance was due to random error (i.e., the difference between 1.000 and 0.917).

## Discussion

Our data replicated Cyders et al.’s. (2014) in terms of factor structure and reliability of the SUPPS-P, in spite of having involved a sample from a different culture that varied more in terms of age and socioeconomic status. As found by Cyders et al. (2014), the model with all SUPPS-P items loading onto a single “impulsivity” factor (Model 1) fit the data poorly, whilst the solution with five distinguishable impulsivity domains (Model 2) and the hierarchical Model 3 had adequate psychometric properties. Additionally, the restrictions imposed from Model 2 to Model 3 did not worsen fits here, nor in Cyders et al.’s. (2014) publication, even when we used a more stringent approach to compare these models (Meade et al., 2008). Hence, regarding Model 3, we confirm (Cyders and Smith, 2007; Cyders, 2013; Berg et al., 2015) that there is a particular association between Positive and Negative Urgency, and Premeditation and Perseverance (see also Billieux et al., 2012).

We used Model 2 instead of Model 3 to analyze if there were any items that were answered differently (DIF) according to age, sex and socioeconomic status and to assess population heterogeneity according to these demographic variables. This choice was based on the following reasoning: (1) Model 2 exhibits direct association between the items and their respective domains (reflected via factor loadings), whereas these relationships cannot be directly tested in the second-order model because the specific factors of each domain are represented by disturbances of the first-order factors (Chen et al., 2006); (2) Model 2 informs in more detail on factors that influence more dissociable impulsiveness domains; 3) Model 2 has also been found in other studies using the full UPPS-P scale with adolescents (Gunn and Smith, 2010) and the UPPS without considering Positive Urgency (e.g., Whiteside and Lynam, 2001; Smith et al., 2007).

In our sample, we found that when five different facets of impulsiveness are considered separately (Model 2), the majority of SUPPS-P items functioned comparably across ages, sexes and different socioeconomic backgrounds. Such inspection is fundamental to assert about the usefulness of comparing groups of individuals with different demographic characteristics in simple statistical analyses such as correlations and *t*-tests. Only three items were answered differently by men and women, reflecting intuitive sex differences: men wanted to fly airplanes to a greater extent, were less bothered by unfinished tasks and reported worrying people more over impulsive behaviors when in unusually positive states. The latter item was also reported more often by younger individuals, which makes sense as they have adult guardians who are responsible for their wellbeing. These effects may be culturally driven or might be found irrespective of respondents’ origins (biological based) (see Cross et al., 2011). Because this type of analyses has not been previously done in the SUPPS-P literature, we have no data do compare our results with.

The finding that sex influences responses in some items is in accord with the sensitivity of Model 2 to population heterogeneity. Males reported lower levels of Premeditation, Positive and Negative Urgency, effects that are not in total agreement with data from Cyders (2013), who found that, among undergraduate North Americans, males indicated higher positive urgency and sensation seeking than females. This may be accounted for by the demographic and/or cultural differences in our and their samples. Another possibility is that our samples varied in terms of genetics and the environment, both of which influence impulsivity (Bezdjian et al., 2011). In fact, meta-analytic and nation-wide surveys often report that sex effects are variable and not always found (Cross et al., 2011; Chamorro et al., 2012; Sharma et al., 2014). Furthermore, some impulsive traits are systematically influenced by regional (individualism–collectivism) and individual cultural differences (independent –interdependent self-concept) (e.g., Kacen and Lee, 2002). Factors that determine an internationally found bias in female volunteering (e.g., Wilson, 2012) must also be considered, as most of our sample was composed of women.

The present study was not designed to investigate factors that could account for possible sex effect and there is little information on how culture affects the SUPPS-P, which is a relatively new scale. Rather, we focused on showing whether this short scale was adequate for use in populations with varied demographic characteristics from non-developed cultures. This was confirmed.

The finding that increases in age were associated to a fall in impulsiveness in all domains except Premeditation corroborates that most aspects of impulsivity decrease throughout adulthood (Reimers et al., 2009; Chamorro et al., 2012), irrespectively, it seems, to country of origin. However, there are exceptions. In the cross-cultural study by Adrianson et al. (2013), despite similar levels of impulsivity (Barratt Impulsivity Scale) in samples from Indonesia and Sweden and higher overall impulsiveness in younger individuals, younger Indonesians had higher impulsivity than older individuals from their culture, while the opposite pattern was found in the Swedish sample. A myriad of socio-cultural differences can be used to explain these differences (see Adrianson et al., 2013) and indicate that age-effects in impulsiveness are influenced by the environment in which people are raised.

The lack of effects of socioeconomic background is of interest, especially as Brazil has a much wider range of social strata than most countries in which the UPPS-P was tested. Low socioeconomic status has been found to be associated to higher impulsivity (e.g., Reimers et al., 2009; Chamorro et al., 2012), but this is difficult to tease apart from biological, environmental and/or cultural differences among samples of different nations, as outlined above. The self-report nature of the SUPPS-P might also explain these results, because socioeconomic status-induced effects on impulsive-like traits are higher when impulsiveness is assessed by others, such as parents or teachers (see Piotrowska et al., 2015).

Overall, we found that, in terms of factor structure, the SUPPS-P behaved very much as it did in the North American undergraduate sample of Cyders et al. (2014). Nevertheless, Shokri and Sanaeepour (2016), who applied this scale in a sample of Farsi speaking undergraduates, only found a 5 domain factor solution after some adjustments to their statitical model. It is possible that this occurred because their study was underpowered due to a small sample or that the adaptation of the scale was not adequate. Given our data, it is unlikely that the necessity of this adjustment stemmed from different socioeconomic status between the North American and Iranian samples. However, other genetic, environmental and/or cultural differences that are not observed when comparing Brazil and the United States, both Western cultures, might be to blame (see Kacen and Lee, 2002). Understanding the reasons why the SUPPS-P behaved differently in Shokri and Sanaeepour’s (2016) study may be possible when further cross-cultural studies on the SUPPS-P are carried out.

The structural similarity of impulsiveness as measured by the SUPPS-P in prior and the present study suggests that these traits reflect expressions of biology, such as genetic predisposition (see Kreek et al., 2005; Fineberg et al., 2014), as observed for personality (see McCrae et al., 2000). However, these characteristics can be modulated and moderated by culture, in that they can result from varying expectations from distinct environmental settings. This can explain part of the different patterns of effects of sex (Cross et al., 2011) and age (e.g., Adrianson et al., 2013) in different samples. The present study, however, was not a cross-cultural investigation in the sense that it did not compare data of different cultures directly. Rather, it descriptively compared the factor structure of the SUPPS-P in a Brazilian and North-American sample. To confirm the extent to which the 5 types of impulsiveness proposed in the SUPPS-P are due to biology and how other socio-cultural factors influence them, cross-cultural investigations must be carried out, ideally investigating genetic and longitudinal maturational changes in the factor structure of the SUPPS-P as done when analyzing personality traits (e.g., McCrae et al., 2000).

In the present study, despite the good reliability of the 5 factor solution and the sensitivity of the different domains to age and sex, under the bifactor model the viability and reliability of using raw scores (participants responses, and not latent traces) on the 5 subscales were poor (with exception of the subscale Sensation Seeking). Moreover, when ω_H_ was compared with Lucke’s ω, around two thirds of the reliable variance in raw scores could be attributed to a general impulsiveness factor determined by adding scores of all items, which reflects individual differences in impulsivity considered only as a broad concept. Importantly, in this case, there was only 8.3% of variance due to random error, which shows that the items adequately captured the intended construct when considering the general concept of impulsivity. Therefore, only around one third of the reliable variance in the raw scores could be associated to the dimensionality of the 5 specific domains. This means that the raw scores obtained in the 5 domains of the SUPPS-P should not be used out of the structural equation modeling environment to indicate different aspects of impulsiveness.

Many factors lead us to believe that the SUPPS-P is a reliable instrument that can be used in different cultural settings: (a) its psychometric properties in a North American (Cyders et al., 2014) and Brazilian samples were similar to each other and to the full UPPS-P scale in a young sample (see Gunn and Smith, 2010) regarding the existence of 5 distinguishable impulsivity dimensions; - like others (Cyders and Smith, 2007; Cyders, 2013; Berg et al., 2015) we also found an association between Positive and Negative Urgency, and Premeditation and Perseverance in a hierarchical model-; (b) it allows a quick characterization of different types of impulsiveness because it involves a significant gain in time of response compared to the full scale (Cyders et al., 2014); (c) sex and variable ages differently influenced distinct domains, making the scale useful to differentiate traits associated to impulsiveness in various cultures, especially as socioeconomic status did not influence results. However, the above mentioned issues regarding the separability of the 5 domains should be considered only *in structural equation modeling environments* and do not hold true when using raw scores, directly derived from respondents’ answers in each domains. This is so because only one third of the reliable variance in the bifactor model could be attributed to the dimensionality associated with the 5 impulsivity dimensions. In contrast, using measures obtained from all items considered jointly (such as adding the raw scores) reliably reflected a general factor of impulsiveness.

We conclude that the SUPPS-P has good psychometric potential and can be useful, in structural equation modeling environments, to investigate 5 distinct impulsiveness domains in populations other than young, highly educated adults from developed countries. However, using raw scores only provides reliable information on a general impulsiveness trait. Future studies must analyze the psychometric properties of the SUPPS-P in clinical populations and elderly individuals. A comparison of the psychometric properties of the short version used here to that proposed by Billieux et al. (2012), despite the shortcomings of the latter (explained in Cyders et al., 2014), would also be of use to the international literature to inform on which short scale best reflects the full UPPS-P factor structure.

## Author Contributions

SP conceived the study, performed the formal analysis, wrote the initial draft, supervised, administrated the project, and acquired the funding. LI, GZ, and RdF performed the experiment and collected data, prepared visualization/data and manuscript presentation, and reviewed the manuscript. LM-D critically reviewed, commented, and revised the manuscript. HC-M performed formal analysis, critically reviewed, commented, and revised the manuscript.

## Conflict of Interest Statement

The authors declare that the research was conducted in the absence of any commercial or financial relationships that could be construed as a potential conflict of interest.

## Footnotes

1 https://www.ncbi.nlm.nih.gov/mesh/?term=impulsivity
